# Editorial: Robots for learning

**DOI:** 10.3389/frobt.2022.1050658

**Published:** 2022-10-21

**Authors:** Wafa Johal, Tony Belpaeme, Mohamed Chetouani

**Affiliations:** ^1^ School of Computer Science and IT, FEIT, University of Melbourne, Melbourne, VIC, Australia; ^2^ Faculty of Engineering, University of New South Wales, Sydney, NSW, Australia; ^3^ IDLab-imec, Ghent University, Ghent, Belgium; ^4^ Centre for Robotics and Neural Systems, University of Plymouth, Plymouth, United Kingdom; ^5^ Institute for Intelligent Systems and Robotics, CNRS UMR 7222, Sorbonne University, Paris, France

**Keywords:** robot for education, learning with robots, human-robot interaction, personalised learning, interactive learning

## Introduction

In the last decade the interest in applying Human-Robot Interaction research to education and learning has boomed. While robots were traditionally used to enthuse learners about STEM subjects, it is now clear that robots can serve to teach subjects beyond STEM, such as languages or handwriting. Researchers have investigated the potential of robots to act as learning or teaching companions for children in classrooms or at home, for elderly people to help maintain their cognitive and physical abilities, and for learners with deficiencies by adapting content to their capabilities. Similarly to Intelligent Tutoring Systems (ITS), robots could have the potential to improve learning gains by personalising content and delivery to the learner. Beyond ITS, robots have a physical presence and can be used for kinaesthetic interaction and embodied learning. Finally, the social capabilities of certain robots could be used to provide adaptive empathic feedback and to engage the learner and to motivate her during the learning task.

While robots for learning is an applied topic of HRI, the context of learner-robot interaction is one of the most challenging and interesting for HRI research. Indeed the research in robots for learning often requires us to work with challenging populations (e.g., children, people with disabilities), it also requires challenging technical integration, and has very clear performance outcomes (i.e., learning gains). In some settings it even requires us to address robot-group interaction, autonomous decision making, joint attention, affective computing. Aiming to go beyond individual interfaces or projects, this Research Topic aimed to attract contributions that enable the generation of guidelines and principles for the design of learner-robot interaction.

This Research Topic focuses on social robotics research, showcasing novel algorithms and computational modeling that are applied within the context of learning. Special focus was given to contributions proposing novel theories, models, and methods for learning with robots. We also welcomed original technical contributions presenting technical systems, algorithms, and computational methods that are tailored to learner-robot interaction. In particular, we were interested in contributions demonstrating the specificities of learner-robot interaction compared to classical human-robot interaction systems.

## Research Topic formation

This Research Topic emerged from a series of workshops and research projects involving the editors, Dr Wafa Johal, Professor Tony Belpaeme and Professor Mohammed Chetouani. Each in our own way, we were drawn to the challenge and promise of using robots in education, and were united in our fascination for the subject and potential to be one of the most impactful applications of social robotics.

## Contents of the Research Topic

### Design of robots for learning

Several stakeholders can be involved in the design of robots for learning: students, their parents, teachers or even organisations. In Lin et al., the authors look into the attitudes of parents towards storytelling robots for children. They present the challenges and opportunities of robots as storytelling companions and highlight the importance of well-being and attention for quality of life of parents when designing robot companions for their children. Johal et al. envision the role of social drones in education taking the perspective of students. They found novel opportunities for drones to potentially support group work but also several challenges that should be addressed in future research on social drone in education. In Smakman et al., the authors interviewed school teachers to examine whether social robots in primary education might compromise the social-emotional development of children, a fear often expressed by teachers and parents who have no experience with robots. Their studies indicate that robots pose little threat, and that on the contrary they might be a preferred educational technology for children with special needs.

### Effectiveness and affectiveness in robots for learning

A key aspect of any educational technology is its impact on learning and learners. Not only are they designed to support teachers, but they need to show their effectiveness in allowing students to learn. In their paper, Kanero et al. use both affective and learning measures to evaluate whether a robot presented over video or a disembodied voice would be more effective at teaching vocabulary in a second language, but found no significant difference. Fischer et al. evaluated whether a robot’s speaking style during second language learning mattered and found that a charismatic delivery resulted in increased learning gains.

### Robots for physical learning

The fact that robots are physical devices has long been touted as important to their use in education. Most often reference is made to their tangible social presence, but robots can also offer a physical experience without necessarily offering a social experience. Kianzad et al. show how physical robots can be deployed in education and present a framework dubbed Physically Assisted Learning (PAL) for learning through haptic support.

### Individual differences in learning with robots

Several studies found that there are significant individual differences in the way students interact and learn with robots. Song et al. report that the level of expertise of the learners (novice or advanced) influenced the potential role and task of a robot supporting the learning of a musical instrument. van den Berghe et al. found in their study that the background knowledge of students influenced their learning experience with a robot tutor. Tolksdorf et al. studied the influence of shyness on robot-learner interaction and found that shyness influenced the affective and the learning aspects.

### Co-learning and adaptation in learning and teaching

Real-time adaptation is also crucial in educational settings. Lee et al. investigated the use of Inverse Reinforcement learning for robots to become better teachers, while Van Zoelen et al., studied how robots could adapt to become better co-leaner in a human-robot teamed search and rescue task.

## Conclusion

Robotics in education, while new, offers plenty of scope and opportunity, which results in a very broad, diverse and rewarding research landscape. Testament to this are the papers collected in this Research Topic. The results presented show that there still is scope to further explore the pedagogy of using robots in education: a one-size-fits-all approach is unlikely to be effective, but how the robot should tailor its responses to maximise the learner’s well-being and learning gains still remains an open question. Beyond the how and why of introducing robots in education, there remains the formidable technical challenge of building a concerted interactive experience with robots. From building robots that can robustly operate in the challenging environment of the classroom, to robots that automatically generate responses that empower the learner, the technical challenges abound. However, the promise and potential of robots in a supporting role are significant and well warrant the formidable research efforts of which this collection is but a sample.

